# Modulation of GSNOR activity for improved NO homeostasis and flood resilience in plants

**DOI:** 10.1080/15592324.2026.2616544

**Published:** 2026-01-15

**Authors:** Thea Wulf, Felix Lutter, Vajiheh Safavi-Rizi

**Affiliations:** a Institute of Biology, Department of General and Applied Botany, Leipzig University, Leipzig, Germany; b Institute of Biology, Department of Plant Physiology, Leipzig University, Leipzig, Germany

**Keywords:** Nitric oxide, *S*-nitrosoglutathione, *S*-nitrosoglutathione reductase, hypoxia, post-translational modifications

## Abstract

Flood-induced hypoxia (low oxygen concentration) is increasing in frequency and intensity due to climate change, leading to significant crop yield losses and posing a major threat to global food security. *S*-nitrosoglutathione reductase (GSNOR) is a highly conserved, cysteine-rich homodimer that regulates the cellular level of the most abundant nitric oxide (NO) reservoir *S*-nitrosoglutathione (GSNO). GSNOR plays a fundamental role in NO homeostasis, as well as in plant development and stress responses, particularly hypoxia. This review summarizes the critical position of GSNOR in the plant hypoxia regulation network. We discuss how GSNOR controls the intracellular pool of *S*-nitrosothiols (SNOs), especially GSNO, thereby mitigating cytotoxic nitrosative stress while fine-tuning NO-mediated posttranslational modifications (PTMs), such as *S*-nitrosylation. Furthermore, we explored the regulation of GSNOR activity through various mechanisms, including oxidative PTMs and protein‒protein interactions. Targeted manipulation of GSNOR activity represents a promising strategy for enhancing flood tolerance in agriculturally important crops. We propose possible approaches for GSNOR manipulation and highlight urgent questions that must be addressed in future research to improve flood resilience in agricultural systems and protect global food security.

## Introduction

Flooding is one of the major threats to global food security and the agricultural sector.^1^ According to the FAO, between 2006 and 2016, flooding events accounted for the most substantial losses in crop yields worldwide​.^2^ With climate change amplifying the intensity of extreme weather events, floods are expected to occur more persistently in the future.^3‐5^ This endangers global food security, particularly as the world population is projected to increase by 26.6% until 2080.^6^^,^^7^ In 2021 38.5% of the global workforce was employed within the agrifood system, highlighting the societal and economic impact of flooding.^8^^,^^9^ Thus, developing innovative strategies to support farmers and ensure food security is an urgent priority.^6^^,^^10^ Developing flood**-**tolerant cultivars is of major importance and although there have already been examples of natural genetic variation such as *SUBMERGENCE 1* (*SUB1*) genes in rice,^11^ translating these results into a wide range of crops is challenging and requires further investigations.

Under adequate oxygen concentration (normoxia, ~20.95% O₂), plants rely on aerobic respiration, while flooding-induced hypoxia (~1–5% O₂), leads to the disruption of main physiological and metabolic processes.^12‐14^ Hypoxia can also occur developmentally, as part of physiological development supporting growth and differentiation, for instance, in bulky organs like potato tubers, in metabolically active tissues such as the phloem, and in developing structures like pollen and lateral root primordia.^14^ How plants respond to flooding is determined by various factors, such as severity and duration of flooding as well as species-specific traits involving signaling, morphological, physiological and molecular adjustments.^12^^,^^14^^,^^15^ Plants employ two major strategies in response to flooding: escape- and quiescence strategy. Escaping includes the elongation of submerged organs, aerenchyma formation, and modifications of leaf anatomy to restore contact with air and light. Species such as *Rumex palustris* and some rice varieties utilize escape strategies.^16^^,^^17^ In contrast, plants experiencing prolonged and complete submergence often adopt a quiescent strategy, suppressing growth to conserve energy until oxygen availability improves. These contrasting responses are underpinned by transcriptional and metabolic reprogramming aimed at sustaining ATP production and minimizing cellular damage.^12^^,^^14^^,^^15^ Other flood-tolerant rice varieties, often called “Scuba rice,” rely on a quiescence strategy. In these plants, the *SUBMERGENCE 1 A* (*Sub1A*) gene limits elongation during submergence, helping to conserve carbohydrate reserves so that the plant can regrow once the water recedes.^18^ Nitric oxide (NO), a small, multitasking signaling molecule, is one of the major players involved in the hypoxia response, developmental processes and response to other biotic and abiotic stresses.^19^ NO regulates a wide range of physiological and molecular processes during hypoxia.^20^^,^^21^ Low levels of NO help plants cope with stress, but when NO accumulates excessively, it triggers the formation of reactive nitrogen species (RNS), leading to nitrosative stress, cellular damage, and ultimately cell death.^22‐24^ NO homeostasis and mitigation of its toxic effects depend on the plant’s scavenging systems, such as phytoglobins (PGBs) and *S*-nitrosoglutathione reductase (GSNOR). PGBs are globular plant proteins that contain a heme group, enabling them to take part in both oxygen and NO metabolism.^25^ When plants experience hypoxia, oxy-phytoglobin helps to convert NO into nitrate, a process that is vital for keeping the cell’s redox balance and energy levels stable. This reaction is a central part of what is known as the PGB–NO cycle.^26^ GSNOR is a highly conserved enzyme that catalyzes the NADH-dependent reduction of *S*-nitrosoglutathione (GSNO) to oxidize glutathione (GSSG) and ammonium.^27‐29^ By regulating GSNO levels, GSNOR controls redox-dependent signaling and NO-mediated posttranslational modifications (PTMs).^25,28,30‐32^ While the significance of GSNOR in maintaining NO homeostasis is well established, its specific function in the hypoxia response remains poorly understood.^33^ Targeted manipulation of GSNOR and related enzymes offers a promising strategy for developing flood tolerance in crops, which could contribute to future food security.^34‐36^ This review provides an overview of the role of GSNOR in NO homeostasis and the hypoxia response in plants. We discuss the mechanisms by which GSNOR activity is regulated and how it contributes to NO-mediated gene regulation and PTMs affecting plant survival under hypoxia. Moreover, we suggest possible strategies for GSNOR manipulation to increase flood tolerance in crops with potential applications. We also address the current open questions regarding the role of GSNOR which remain to be explored, to advance the framework for improving resilience in agricultural systems and protecting global food security.

## Role of NO under hypoxia

The functions of NO in response to hypoxia and flooding range from the formation of morphological adaptations such as aerenchyma and adventitious roots to switching towards anaerobic metabolism.^21^ NO modulates stomatal conductance and transpiration under flooding conditions, and more broadly, hypoxia-released NO increases the expression of genes related to phytohormone signaling, transcription factors (TFs) involved in hypoxia sensing, stress responses, and reactive oxygen species (ROS).^20^

One of NO’s most critical functions is its involvement in oxygen sensing of plants through the Plant Cysteine Oxidase (PCO)-*N*-degron-pathway, which regulates the stability of subgroup VII of Ethylene Response Factors (ERFVIIs).^12^^,^^15^ Under normoxic conditions, NO and oxygen promote the oxidation of the *N*-terminal cysteine (Cys) of ERFVIIs, targeting them for proteasomal degradation.^37^^,^^38^ Under hypoxia, ERFVIIs are stabilized and translocated into the nucleus,^39^ where they activate the transcription of hypoxia-responsive genes (HRGs).^40^ This response is supported by ethylene which leads to the expression of *PHYTOGLOBIN 1* (*PGB1*).^37^^,^^41^ Phytoglobins scavenge excess NO, resulting in the stabilization of ERFVIIs.^37^^,^^41^ Beyond transcriptional regulation, NO influences mitochondrial function. Early work by Stoimenova et al. ^42^ demonstrated that the mitochondria of anoxia-tolerant rice cultivars produce more NO under hypoxia compared to intolerant barley cultivars.^42^ These findings suggest a role in stress resistance. NO can regulate the respiratory chain and is therefore responsible for maintaining steady oxygen levels. NO formed from nitrite in the mitochondria reversibly inhibits cytochrome c oxidase (COX).^43^ This NO–nitrite feedback loop supports not only hypoxia tolerance but also germination and growth under stress conditions.^43‐46^ Additionally, NO promotes antioxidant activity, mitigating ROS accumulation and protecting cell structures, including DNA.^47^ Excessive amounts of NO shift its function from a signaling molecule to a cytotoxic agent, inducing nitrosative stress, which can lead to programmed cell death (PCD).^22^^,^^24^^,^^48^ Hence, the regulation of NO levels is vital, with key control points residing in mitochondrial enzymes such as COX, as well as in the PGB-NO cycle mentioned earlier. As part of the PGB-NO cycle, NAD^+^ is produced, which promotes anaerobic metabolism under hypoxia.^26,49‐53^ NO not only regenerates redox equivalents but also alters metabolism toward fermentation by inhibiting COX and directly or indirectly upregulating genes responsible for fermentation and glycolysis.^20^^,^^43^ NO can activate alternative oxidase (AOX), which generates more NO feeding into the PGB-NO cycle.^54^^,^^55^ Therefore, NO functions as both an oxygen sensor and a metabolic switch during hypoxia, stabilizing ERFVIIs while altering mitochondrial and redox metabolism. Beyond these roles, NO facilitates different PTM that alter protein function,^56^^,^^57^ thereby setting the stage for its broader role in stress regulation.

## NO bioactivity and PTMs

A range of PTMs involved in both physiological and stress responses are regulated by NO.^57^ NO-dependent PTMs can occur through two mechanisms: direct, where NO or its derivates covalently modify specific protein residues or metal centers, and indirect, where NO modulates upstream proteins or signaling cascades, which induces more PTMs.^56^^,^^57^ NO mediated PTMs include metal nitrosylation, tyrosine (Tyr) nitration, and *S*-nitrosylation, the latter being important for hypoxia-related signaling.^33^^,^^56^^,^^58^ *S*-nitrosylation is the covalent attachment of an NO moiety to a Cys residue, forming *S*-nitrosothiols (SNOs).^59^ The modification can alter enzymatic activity, conformation, or interaction with target proteins.^56^ As a reversible modification that happens under regulated cellular conditions, *S*-nitrosylation is well suited for fast changes in stress signaling.^56^^,^^60^^,^^61^ Under hypoxic conditions NO exerts its regulatory effects through direct and indirect target *S*-nitrosylation. It modifies proteins that in turn influence pathways such as ubiquitination and arginylation in the *N*-degron pathway. These modifications regulate protein stability and are essential for adaptation to environments with low oxygen concentrations. NO can affect arginine methylation of stress-related proteins and modulate histone acetylation, impacting gene expression under abiotic stress.^57^ There is also evidence for direct effects of NO through *S*-nitrosylation during hypoxia.^57^^,^^62^ *S*-nitrosylation controls the detoxification function of PGB1 during hypoxic stress and enhances the enzymatic activity of ascorbate peroxidase (APX).^63^

NO-regulated PTMs result in functional changes to target proteins, making control of NO signaling essential. Despite NO being the primary signaling radical, due to insufficient electrophilicity it does not react directly with cysteine thiols to form SNOs under physiological conditions.^56^ Instead, *S*-nitrosylation requires the oxidation of NO to higher oxides (e.g., N_2_O_3_) or nitrosonium cations (NO^+^) or the involvement of radical intermediates.^56^^,^^64^ Thus, most protein *S*-nitrosylation within the cell is mediated by SNOs acting as transnitrosylating agents, primarily GSNO, the *S*-nitrosylated form of glutathione (GSH).^56^^,^^59^^,^^64^

## Chemistry and stability of GSNOR as a NO donor

GSNO functions as an intracellular SNO, bioavailable RNS reservoir, and transnitrosylating agent.^59,64‐66^ To enable functional stress adaptation, its intracellular concentration is closely regulated by both biotic and abiotic stimuli.^67^^,^^68^ Therefore in plants, GSNO is regulating the SNO pool and fine-tunes NO signaling.^69^ GSNO itself is considered as a primary SNO, with higher stability compared to other SNOs.^64^ It can be formed through three reactions: (1) reaction of GSH or GS^-^ with oxidation products of NO, such as N_2_O_3_, (2) radical coupling between GS∙ thiyl radical and NO, and (3) reaction as between GSH and nitrite under acidic conditions or with nitrous acid.^64,70‐72^ The reaction of a GSNOH radical intermediate with an electron acceptor like O_2_ to form GSNO seems to be of minor significance.^71^

Under physiological conditions, the spontaneous thermal homolysis of S-NO from GSNO, releasing NO, does not occur. The cleavage requires external factors such as UV light or metal ions.^64^^,^^73^ GSNO can be reduced by GSNOR, producing an unstable *N*-hydroxysulfinamide intermediate that spontaneously rearranges and decomposes into oxidized forms of GSH (e.g., glutathione sulfinic acid) and ammonia. Alternatively, the unstable intermediate can react with GSH to form GSSG and hydroxylamine.^70^^,^^74^^,^^75^ Ultimately, GSNO is broken down into end products such as ammonia and hydroxylamine, which can no longer *S*-nitrosylate cellular targets. GSNO is a uniquely stable yet biologically reactive SNO species. Owing to its chemical properties, it acts as an important physiological reservoir and carrier of NO bioactivity. However, to prevent uncontrolled nitrosative signaling, GSNO levels must be tightly regulated—primarily through the activity of GSNOR.

## GSNOR-mediated NO homeostasis

In plants, GSNOR was first isolated and described in *Pisum sativum* as a formaldehyde-active class III alcohol dehydrogenase.^29^ Later, in studies on Arabidopsis, it was identified as a GSH-dependent formaldehyde dehydrogenase (GS-FDH).^76^ GSNOR is a conserved protein that regulates NO homeostasis across all kingdoms.^28^ In 2002, Sakamoto et al. showed that GSNOR functions as an *S*-nitrosoglutathione reductase via the heterologous expression of Arabidopsis *GS-FDH* in *Escherichia coli*.^77^ These discoveries, particularly the functional identification in Arabidopsis, confirmed the role of GSNOR as a regulator of NO homeostasis.

In Arabidopsis, there is only a single-copy gene of GSNOR (*AtGSNOR1*), which was formerly known as *SENSITIVE TO HOT TEMPERATURE* (*HOT5*) and *PARAQUAT RESISTANT2* (*PAR2*).^30^^,^^32^ The single-copy gene in Arabidopsis is a valuable model for reverse genetics studies, since any defects observed are more directly attributable to its lack of function. Structurally, GSNOR is a Cys-rich homodimer and catalyzes NAD^+^/NADH-dependent oxidoreductase activity using zinc as a catalytic and structural cofactor.^78^^,^^79^ GSNOR consists of nicotinamide cofactor-binding and catalytic domains, with zinc-coordinating residues highly conserved among green plants.^78^^,^^79^ Among its 15 Cys residues, nine of them are extra nonzinc-coordinating Cys residues (exzinc Cys).^79^^,^^80^ Most of the exzinc Cys residues of GSNOR in Arabidopsis were found to be inaccessible to solvents, hinting at a structural role. However, three of these Cys (Cys-10, Cys-271, Cys-370) were determined to be solvent accessible and would therefore be available for PTMs.^79^ GSNOR is broadly expressed in Arabidopsis, with GSNOR-GFP fusions detected throughout seedlings and floral tissues.^79^ Subcellular localization studies revealed that GSNOR-GFP is mainly distributed in the cytosol and nucleus (excluding the nucleolus).^79^ Additionally, in pea leaves, immunogold-labeling studies demonstrated the presence of GSNOR in multiple organelles, including the cytosol, chloroplasts, mitochondria and peroxisomes.^81^ This widespread localization is consistent with its central role in maintaining intracellular NO and SNO homeostasis.

GSNOR-mediated control of NO homeostasis is fundamental to a broad array of physiological processes, particularly during biotic and abiotic stress responses. By fine-tuning the intracellular NO and SNO levels, the enzyme prevents nitrosative stress while preserving the NO signaling capacity. Its role has been documented across various species and stresses, including iron (Fe) and aluminium (Al) toxicity, hypoxia, salinity, waterlogging (WL), and pathogen attacks.^82‐88^ In root meristems, GSNOR prevents NO-induced cell death.^87^ In reproductive tissues, it maintains fertility by regulating *S*-nitrosylation patterns that are essential for protein quality control and hormone signaling.^85^ The highly conserved structure of GSNOR across green plant species and its localization in various cellular compartments underscore its important role in NO homeostasis and redox balance. Consistent with this fundamental function, genetic approaches have proven highly effective in demonstrating the role of GSNOR in stress responses. Accordingly, GSNOR mutants are valuable for elucidating the physiological and molecular consequences of compromised GSNOR activity.

## GSNOR mutants

In Arabidopsis, *GSNOR* loss-of-function mutants such as *hot5-2* (also known as *gsnor1-3*/*par2-1*) and *hot5-4* exhibit null alleles with undetectable enzymatic activity.^30‐32,89,90^ This results in developmental defects, hypersensitivity to stress, and altered de-nitrosylation dynamics.^89^ Mutant phenotypes underscore the critical function of GSNOR in sustaining basal NO levels, which are required for viability. In the *hot5-2* mutant, the abundance of proteins from the aldo-keto reductase (AKR) family was highly upregulated.^91^ AKRs are induced upon various stresses, including hypoxia, suggesting that they might act as a compensatory mechanism for degrading GSNO in GSNOR-deficient plants. They primarily function as general oxidoreductases with broad substrate specificity, treating GSNO as one of many reactive substrates.^91^ Therefore, GSNOR probably remains the main pathway for NADH-dependent GSNO degradation in nondeficient plants. Partial-function mutants such as *hot5-3* and *hot5-1*, which have some residual GSNOR activity, reveal that GSNOR's regulatory role in NO signaling is not simply on or off, but likely depends on a threshold level of enzymatic activity, below which NO accumulates to harmful levels.^32^ The fact that both mutants exhibit heat sensitivity only in the dark, and not in the light, underscores the context-dependent nature of NO detoxification requirements, likely reflecting differences in redox balance, developmental signaling, or photoreceptor-mediated modulation of stress pathways.^32^ Complementation studies by Sun et al. ^84^ on *gsnor1-3* with Cys-modified GSNOR (*GSNOR1C370S-GFP*) alleles further emphasized the enzyme's nuanced regulatory role.^84^ While mutation at Cys-370 restores normal growth, modification of Cys-284 leads to reproductive defects, suggesting that site-specific PTMs (e.g., *S*-sulfenylation) have a critical influence on developmental signaling.^84^ Whereas GSNOR null mutants suffer from excessive NO accumulation leading to an impaired stress response and developmental abnormalities, overexpression lines with significantly lowered SNO levels display altered shoot architecture, delayed flowering, and reproductive defects.^31^^,^^89^ Even partial activity loss or mis regulation at a single residue can disturb NO signaling critical for normal development and stress resilience. In addition to Arabidopsis, single knockout studies on the two GSNOR gene copies in *Lotus japonicus* revealed species-specific phenotypes, including impaired nodulation and delayed flowering, but less severe developmental arrest, possibly due to the two copies of the gene.^92^^,^^93^ However, while both enzymes display similar GSNO reductase activity, they differ in their tissue expression profiles, which means that they are not completely redundant.^93^ CRISPR/Cas9-generated *osgsnor* of *Oryza sativa* (rice) mutants exhibit root growth inhibition and hypersensitivity to Al stress.^94^ In *Solanum lycopersicum* (tomato), *SlGSNOR* knockdown results in lethality when protein reduction exceeds ~60%, accompanied by severe defects in seed viability, fruit development, and pathogen resistance.^95^ In contrast, overexpressing lines presented increased disease resistance via salicylic acid (SA) pathway activation, albeit with reduced total yield. These findings indicate that both overexpression and loss-of-function mutations disrupt the delicate equilibrium of GSNOR in terms of growth and immunity.^95^ Plant growth and stress tolerance rely on a balanced level of GSNOR activity, disbalancing SNO signaling and leading to species-specific developmental and immune defects. Therefore, GSNOR activity must be strictly controlled to maintain optimal NO signaling and prevent cytotoxic effects.

## Regulation of GSNOR

GSNOR expression is upregulated in response to high salt, Al toxicity, WL and postharvest recovery,^82^^,^^86^^,^^88^^,^^96^ playing a role in the reduction of RNS-induced cytotoxicity. In addition to *S*-nitrosylation, oxidative PTMs such as *S*-sulfenylation also regulate GSNOR activity. A detailed synthesis of GSNOR PTMs is provided in the review by Lindermayr.^97^ For instance, hydrogen peroxide ($H_{2}O_{2}$) inhibits GSNOR1 via *S*-sulfenylation at Cys-284, a redox-sensitive residue.^84^ GSNOR can thus also function as a redox sensor integrating ROS and RNS signals during developmental events such as ovulation and fertility.^84^ This regulatory complexity of GSNOR is further enhanced by nitro-fatty acids, such as nitro-linolenic acid (NO_2_-LN), which influence both its transcriptional and post-translational regulation.^98^ In genotypes deficient in NO_2_-LN detoxification, an accumulation of NO_2_-LN is associated with decreased *GSNOR1* transcript and protein levels. While NO_2_-LN can *S*-nitrosylate in an NO-dependent matter and inhibit GSNOR1 enzymatic activity, it may also alter transcription factor binding to the *GSNOR1* promoter, suppressing gene expression.^98^

Additionally, regulation occurs through nonredox PTMs, such as the reported calmodulin (Cam)-dependent inhibition of AtGSNOR1, which promotes NO accumulation in processes such as salt resistance.^99^ In addition to regulation through PTMs, GSNOR activity is modulated by protein‒protein interactions. Tabassum and Loake^100^ identified thioredoxin (TRX) isoforms as potential regulators of GSNOR activity via denitrosylation. Specifically, TRX *h*-type 5 (TRX*h*5) appears capable of reversing GSNOR *S*-nitrosylation, thus restoring its function. The study demonstrated that mutations in the catalytic Cys residues of either GSNOR or TRX*h*5 inhibited this interaction, suggesting functional coupling between the two proteins. These findings reveal a mechanism in which two major NO-regulatory enzymes coordinate the *S*-nitrosylation status during immune responses. Such interactions may indicate a more generalized strategy for controlling redox signaling networks in plants.^100^ While Tabassum and Loake^100^ identified TRX-*h* as a protein that reverses GSNOR inactivation, there is also evidence that the plant quiescin sulfhydryl oxidase homolog (QSOX1), which contains TRX-like domains, inactivates GSNOR through oxidation.^101^ This occurs as part of a pathogen-induced negative feedback loop (PNFL) aimed at restoring redox homeostasis. Inactivating GSNOR leads to increased SNO levels, which can *S*-nitrosylate and inhibit the NADPH oxidase RBOH, thereby reducing ROS production.^101^ Interestingly, the reduced GSNOR activity by QSOX1 might yield in a higher GSNO level, which is ultimately affecting QSOX1 again.^102^ QSOX1 can be *S*-nitrosylated by the accumulated GSNO, resulting in the formation of oligomers.^102^ Additionally, this oligomerization is inducible by heat shock.^102^ During oligomerization, the function of QSOX1 changes from a thiol-based redox sensor to a molecular chaperone.^102^ *QSOX1* overexpressing and knockout Arabidopsis mutants showed strong resistance and high sensitivity to heat shocks, respectively.^102^ GSNO treatment significantly enhanced the heat shock resistance in the QSOX1 overexpressing line, indicating that these QSOX1 complexes/oligomers are crucial in heat shock resistance.^102^ Furthermore, H_2_O_2_ treatment did not result in an increased oligomerization of QSOX1,^102^ which might imply the distinct roles of NO and H_2_O_2_ for the plant. GSNOR regulation also intersects with nitrate reductase (NR)-derived NO production.^103^ During cold acclimation in tomatoes, NR-dependent NO production and GSNOR activity increase. When NR is silenced, GSNOR activity and NO levels decrease, reducing cold tolerance, while silencing GSNOR suppresses NR but increases NO accumulation, which in turn improves cold tolerance. NR and GSNOR therefore work together to maintain NO homeostasis during cold stress, demonstrating a link between NR and GSNOR.^103^ At the posttranslational level, GSNOR abundance is further controlled by the *REPRESSOR OF GSNOR1* (ROG1), a transnitrosylase that promotes GSNOR degradation and thereby reduces NO detoxification.^104^ Mutants lacking *ROG1* demonstrate increased NO sensitivity, suggesting that *ROG1* functions as a negative regulator of GSNOR-mediated NO homeostasis.^104^ This position *ROG1* is part of a regulatory network characterized by flexibility and feedback control. Lastly, AKRs were identified as alternative enzymes capable of degrading GSNO, especially under conditions where GSNOR is absent or inhibited.^85^^,^^91^ This raises the possibility of functional redundancy or distributed regulation, where multiple systems may contribute to GSNO catabolism depending on the specific stress context, developmental stage, or species-specific response. Together, these regulatory mechanisms, including oxidative and nonoxidative PTMs, transcriptional modulation, and protein–protein interactions, make GSNOR sensitive to changes in the cellular redox status. This multilayered control is particularly important under hypoxic conditions. The following section details how low-oxygen conditions impact GSNOR activity and expression.

## Regulation and expression of GSNOR under hypoxia

One of the earliest consequences of oxygen deprivation is the disruption of the ascorbate‒glutathione cycle (AGC).^105^ In this cycle, ascorbate peroxidase (APX) catalyzes the reduction of H_2_O_2_ using electrons from ascorbate, thereby oxidizing two molecules of ascorbate to monodehydroascorbate (MDA) and producing water.^106^^,^^107^ Two MDA molecules can react with each other in a disproportionation reaction to form one molecule of ascorbate and one molecule of dehydroascorbate (DHA). DHA is reduced by DHA reductase (DHAR), which uses electrons from two glutathione molecules, resulting in oxidized glutathione disulfide (GSSG). Finally, GSSG is reduced by glutathione reductase (GR) using electrons from NADPH. Additionally, two molecules of MDA can be reduced to ascorbate by the MDA reductase (MDAR) by using electrons from NAD(*P*)H.^106^ These reactions maintain the cellular redox buffering capacity, but under hypoxia, their efficiency decreases, leading to the accumulation of ROS. The ROS directly influences the GSNOR function. In Arabidopsis, ROS accumulation induced by the herbicide paraquat decreases GSNOR activity,^33^ indicating that oxidative stress can inhibit this enzyme. Under near anoxic conditions (~0.001% $O_{2}$), NO production in *Nicotiana tabacum* (tobacco) leaves correlates with the transcript amounts of *GSNOR.*^108^ Tobacco *AOX* overexpressing mutant-lines with increased NO emission, showed the highest *GSNOR* transcript amounts during the first 6 hours of hypoxia.^108^ This early activation likely acts as a buffering mechanism to prevent excessive NO accumulation by increasing GSNO turnover.^108^ In wild-type and *AOX* knockdown lines, *GSNOR* expression showed an oscillatory pattern: a marked increase after 6 hours, a decline after 12 hours, and return to induction after 24 hours.^108^ In contrast, *AOX* overexpression lines showed consistently high *GSNOR* transcription levels throughout the 24 hour treatment.^108^ Comparable regulatory patterns related to short-term flooding stress were observed in *Glycine max* (soybean).^109^ Exogenous NO donors (SNP and CySNO) can activate and upregulate the transcription of *GSNOR1* in soybean cultivars at specific short-term flooding phases.^109^ The Daewon (normal) cultivar upregulated *GSNOR1* transcription in later flooding phases (6 h, especially under SNP treatment), whereas the Pungsannamul (sensitive) cultivar showed earlier activation (3 h, under SNP and CySNO treatments).^109^ This lower level of NO-related gene expression in Pungsannamul (sensitive) after 6 h suggests a more vulnerable NO scavenging system that becomes overwhelmed as stress progresses.^109^ Disruption of GSNOR activity under hypoxia has important functional consequences. Reduced GSNOR activity leads to the accumulation of GSNO as well as to increased NO and SNO levels. Elevated NO levels may, in turn, support ROS scavenging, as APX becomes activated through *S*-nitrosylation.^107^ Additional NO might be supplied by xanthine oxidoreductase (XOR), whose xanthine oxidase (XO) isoform reduces nitrite to NO under anaerobic conditions, using NADH as an electron donor,^110^ further reinforcing redox buffering. Under specific conditions, *GSNOR* expression is downregulated. Hesari et al. ^82^showed that in waterlogged *Cucumis sativus* (cucumber) roots treated with nitrate, both GSNOR expression and activity decreased.^82^ In their study, *GSNOR* levels decreased under WL and nitrate treatments but increased when NO was scavenged, indicating that GSNOR inactivation may have occurred through NO-mediated mechanisms. Similar results were reported earlier by Frungillo et al. ^25^, who showed that nitrate treatment reduced GSNOR activity.^25^ In WL and nitrate treated cucumber roots, expression of *CsRAP2.3*, a putative ERFVII-type transcriptional regulator, was further downregulated compared to WL alone.^25^ A candidate *GSNOR* gene in cucumber was induced by WL stress, repressed by combined WL and nitrate treatment, and re-induced when an NO scavenger was applied.^25^ Although this pattern initially appeared consistent with direct NO regulation, the authors proposed that it more likely reflects transcriptional regulation by CsRAP2_3, rather than a direct response to NO concentration.^25^ These findings suggest a regulatory strategy in which the plant permits NO to accumulate under WL, thereby enhancing energy production via anaerobic respiration.^82^

**Figure 1.**
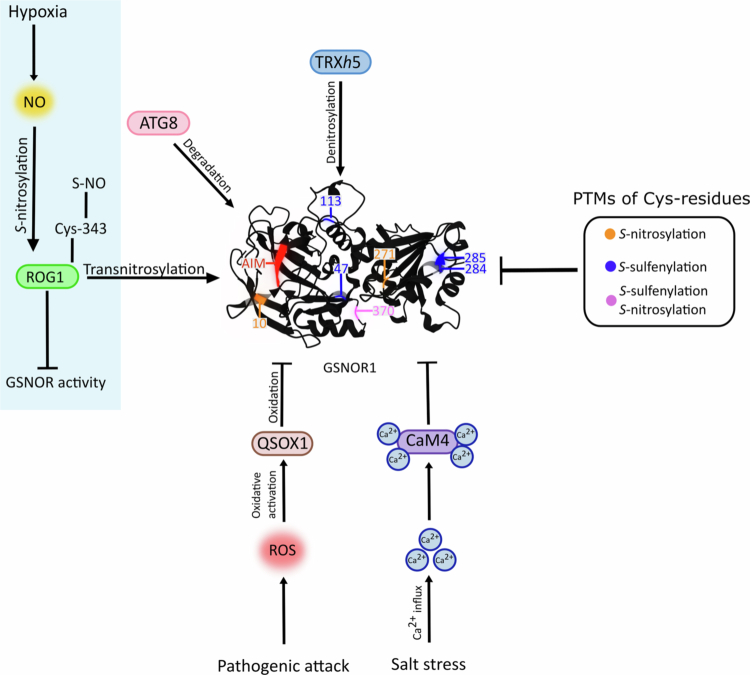
Structure of the GSNOR1 monomer from Arabidopsis and its post-translational regulation. Important (ex-zinc) Cys residues are highlighted in orange, blue and pink to indicate possible *S*-nitrosylation, *S*-sulfenylation modification or both.^33^^,^^70^^,^^84^^,^^111^ Hypoxia leads to *S*-nitrosylation of the Repressor of GSNOR1 (ROG1), which transnitrosylates GSNOR, resulting in its degradation.^104^ In addition, *S*-nitrosylation of Cys-10 and revealing the AUTOPHAGY-RELATED 8-interacting motif (AIM) leads to degradation of GSNOR by Autophagy-related protein 8 (ATG8).^33^ Quiescin sulfhydryl oxidase homolog (QSOX1) oxidizes GSNO, leading to its inactivation.^101^ Salt stress induces a Ca^2+^ influx, which binds to calmodulins (e.g. CaM4), ultimately inhibiting GSNOR.^99^ Thioredoxin (TRX), specifically TRX *h*-type 5 (TRX*h*5), denitrosylates GSNOR and restores its activity.^100^ The protein structure of the GSNOR1 monomer from Arabidopsis was obtained from Alphafold 3.^112^

One of the most striking mechanisms of GSNOR regulation under hypoxia is its *S*-nitrosylation dependent autophagic degradation.^33^ Zhan et al.^33^ demonstrated that hypoxia triggers *S*-nitrosylation of Cys-10 in GSNOR1, inducing conformational changes that expose an otherwise hidden AUTOPHAGY-RELATED 8-interacting motif (AIM).^33^ Exposure of AIM marks GSNOR1 for selective autophagy, reducing its abundance and enzymatic activity.^33^ This negative feedback loop allows NO concentrations to rise, thereby activating NO-dependent signaling pathways critical for hypoxia tolerance. These include the induction of genes for *ADH1* and *PDC*, which are key enzymes in anaerobic metabolism. The degradation of GSNOR1 represents a regulatory mechanism from NO scavenging to signaling, aligning cellular metabolism with hypoxia.^33^ A further study by Chen et al.^104^ identified ROG1 as a potential component of this negative feedback loop. ROG1 mediates the transnitrosylation of NO to Cys-10, potentially reactivating GSNOR as hypoxic conditions are alleviated, suggesting a reversible system for modulating GSNOR activity according to the cellular redox environment.^104^ Plants regulate GSNOR accordingly to respond to hypoxic conditions. As such, targeted manipulation of GSNOR activity through genetic engineering or modulation of its regulatory pattern may offer new strategies to increase plant survival under hypoxia. Figure 1 illustrates the structure of the Arabidopsis GSNOR1 monomer and highlights its posttranslational regulation. Considering the critical roles of GSNOR and GSNO within the hypoxia-responsive signaling network, accurate measurement of GSNO levels as well as GSNOR activity are essential for understanding redox- and NO-dependent physiological responses.

## Measurement of GSNO and GSNOR activity

There are only a few methods available for quantifying GSNO levels, which differ in their selectivity for GSNO.^64,113‐115^ While working with GSNO, it is important to prevent its degradation, as it easily decomposes when exposed to light, heat, reducing agents or Cu⁺.^113^ Because of this, experimental steps should be carried out cold, protected from light and with minimal processing time.^113^ In addition, metal chelators should be added to the sample to prevent metal-catalyzed decomposition of GSNO.^64^ To avoid an artificial increase in GSNO content, acidification of the sample in the presence of nitrite should be avoided, as GSNO can be formed from GSH and nitrite under acidic conditions.^64^ Otherwise, pretreatment is recommended to remove nitrite and block the free thiol of GSH.^64^

The most promising method to quantify GSNO content is the liquid chromatography-electrospray ionization mass spectrometry (LC-ESI-MS) method.^64^^,^^113^ This approach allows simultaneous measurement of GSH and GSSG levels, as demonstrated by Airaki et al. ^113^. For the chromatographic identification of GSNO, the same column and mobile phases can be used as for GSH and GSSG.^113^ However, GSNO has a distinct retention time of approximately 9.05 minutes, which allows for differentiation. The identification of GSNO is based on both retention time and its characteristic mass‒charge transition, which enables clear detection in plant extracts.^113^ In addition, GSNO levels can be measured using gas chromatography‒mass spectrometry (GC‒MS).^116^ Since GSNO is nonvolatile and thermally unstable, it cannot be analyzed directly using GC-MS. Therefore, the SNO group is first converted to nitrite, which is then converted into a volatile derivative that can be detected.^116^ GSNO is first treated with mercury chloride (HgCl_2_), which breaks the SNO bond and releases nitrite. The nitrite is derivatized with pentafluorobenzyl (PFB) bromide, forming a stable, volatile PFB-nitrite, which is extracted in toluene and injected into the GC for analysis. Detection was performed using electron capture negative ion chemical ionization mass spectrometry (GC–ECNICI–MS). Subsequent quantification is based on selected ion monitoring of the fragment ions: m/z 46 for unlabeled nitrite originating from endogenous GSNO, m/z 47 for ^15^*N*-labelled nitrite originating from the internal standard GS^15^NO. The GSNO concentration is calculated from the ratio of these peak areas multiplied by the known amount of the isotope-labeled internal standard.^116^

Other methods for measuring the GSNO content include colorimetric and fluorometric measurements, UV/Vis measurements, and NO release measurements. All these methods are based on the S-NO bond and can detect other SNO compounds in addition to GSNO.^64^^,^^114^ Initial separation of GSNO from the other SNO-containing compounds by high-performance liquid chromatography (HPLC) is therefore essential.^64^ To date, the most reliable colorimetric approach is the Saville assay.^64^^,^^114^^,^^117^ This assay is based on the spectrophotometric measurement of azo dyes. First, SNOs are cleaved by Hg^2+,^ which releases nitrite. Nitrite can then react with an aromatic amine (e.g., sulfanilamide) to form a diazonium salt. This salt can be used to synthesize azo dyes, which are then measured to quantify the SNO content.^64^^,^^114^^,^^117^ *N*-(1-naphthyl)ethylenediamine is commonly used to form azo dyes, which then absorb at 540 nm.^118^ The difference between the absorption at 540 nm with and without Hg^2+^ reflects the level of total GSNO in the sample, given that GSNO is separated from other SNO before the measurements.^114^ If there is no separation enabled beforehand, this approach quantifies the total amount of SNOs. In particular, samples with a high nitrite content can lead to a high background,^64^ thus, separation of GSNO is recommended. A modified version of the Saville assay, in which sulfanilamide and *N*-(1-naphthyl)ethylenediamine were replaced by 2,3-diaminonaphthalene, allows the detection of SNO quantities down to 50 nM.^119^ In this modified version, the species released from SNO reacts with 2,3-diaminonaphthalene to form 2,3-naphthyltriazole.^119^ In addition, GSNO can be measured spectrophotometrically based on the characteristic absorption peak of the S-NO bond in the range of 334–338 nm.^64^^,^^114^^,^^115^^,^^119^ According to the data, the molar extinction coefficient of GSNO is between *ε* = 900–922 m^−1^ cm^−1^.^64^^,^^114^^,^^115^^,^^119^

Determining GSNOR activity can be conducted via UV–visible spectrophotometric assay that monitors the oxidation of NADH in the presence of GSNO.^32^^,^^77^ Because GSNOR catalyzes the reduction of GSNO via oxidation of NADH (or less commonly NADPH).^32^ NADH consumption is measured as the decrease in absorbance at 340 nm, corresponding to its absorption maximum and quantified using its molar extinction coefficient $\varepsilon_{340}$  = 6220 $M^{-1}$ cm^−1^.^32^^,^^77^ By measuring background NADH oxidation in control samples lacking GSNO, enzymatic rates are corrected for non-enzymatic NADH degradation.^32^ The protein activity is normalized to total protein content and typically expressed as nmol NADH consumed min⁻¹ mg protein⁻¹^32^^,^^77^ GSNOR activity can also be visualized after non-denaturing polyacrylamide gel electrophoresis (native PAGE) combined with activity staining.^120^ After electrophoretic separation, gels are incubated in 0.1 M sodium phosphate buffer (pH 7.4) containing 2 mM NADH. The excess buffer is then removed, and the gels are covered with filter paper soaked in 3 mM GSNO. Finally, the filter paper is discarded, and the gels are exposed to UV light to detect GSNOR enzymatic activity bands. Taken together, the methods for quantifying GSNO and evaluating GSNOR activity offer tools to investigate its impact under hypoxia. Combined with studies on GSNOR mutants, such insights establish the foundation for future research aimed at engineering GSNOR activity to improve plant performance during hypoxia.

## Engineering strategies of GSNOR

Loss-of-function of *GSNOR* results in developmental defects, altered stress responses, and reduced fertility or yield.^30^^,^^31^^,^^89^^,^^90^^,^^95^ Although transgenic overexpression of *GSNOR* rescues fertility defects in the *hot5-2* null mutant,^121^ hyperaccumulation of the enzyme can lead to a reduced seed set or total yield compared to wild-type plants.^89^^,^^95^ Hence, breeding of improved hypoxia tolerant crops cannot rely on knockout or overexpression of *GSNOR*. Instead, context-dependent modulation, such as the use of stress-inducible promoters, *cis*-regulatory elements, and tissue-specific expression systems, may provide more precise regulation of GSNOR. A promising concept could involve the temporal adjustment of GSNOR activity to increase hypoxia signaling without causing NO toxicity.^27^^,^^30^^,^^32^^,^^82^^,^^87^ Specifically, temporary inhibition of GSNOR during the early phases of flooding could allow NO signaling, while reactivation during recovery would prevent NO toxicity.^33^^,^^70^^,^^122^ Zafari et al.^108^ demonstrated this transcriptional regulation in Arabidopsis, making it a potential target for improving flood tolerance in crops.^108^ The reengineering of NO-mediated hypoxia signaling pathways is an appropriate strategy and could serve as a useful model for GSNOR-based engineering. For instance, studies on the expression of *MaERFVII3* from *Musa acuminata* in Arabidopsis resulted in improved flooding tolerance.^123^ Similarly, manipulation of PCOs can increase hypoxia tolerance.^34^^,^^36^ While challenges arise when *N*-terminal cysteine oxidases (NCOs) from nonplant kingdoms are used, as they often lack compatibility with native plant systems, targeted engineering within the plant system has yielded positive results. Dirr et al. ^34^ successfully engineered Arabidopsis *PCO4* variants that exhibited reduced enzymatic activity and resulted in improved survival and recovery after submergence.^34^ Nevertheless, this approach must be balanced, as enhanced expression of HRGs due to reduced NCO activity often correlates negatively with development and biomass accumulation under normoxia.^36^ To avoid the limitations of endogenous signaling, orthogonal gene circuits offer a powerful alternative.^35^ These synthetic constructs are designed to operate independently of native pathways, thereby minimizing crosstalk and enhancing predictability.^35^ Oxygen-responsive modules derived from the human hypoxia pathway (PHD-HIF-VHL) have been successfully implemented in Arabidopsis, showing a functional, oxygen-dependent degradation system that can be adapted for plant use.^124^ Future approaches may include the introduction of non-native NO scavengers from other eukaryotic kingdoms, arranged in a cell-type and temporally specific manner. This highly controlled approach is necessary because constitutive manipulation of NO homeostasis frequently leads to pleiotropic developmental defects.^27^^,^^30^^,^^32^ The expression of *GSNOR* under hypoxia ensures that it is capable of mitigating NO-dependent processes.^70^ However, its protein abundance is decreased through PTMs and autophagy, therefore enabling the accumulation of NO and the activation of anaerobic metabolism.^33^^,^^104^ The engineering of GSNOR alleles resistant to degradation, such as mutations at Cys-10, has the potential to extend NO buffering without complete functional loss, offering increased stress resistance during early hypoxic events. Specifically, substitution of Cys-10 with serine makes the protein insensitive to protein degradation while retaining enzymatic activity.^33^ PTMs such as *S*-nitrosylation (Cys-10) and *S*-sulfenylation (Cys-284) modulate the function of GSNOR in response to environmental cues.^27^^,^^33^^,^^84^^,^^97^ *S*-nitrosylation reversibly inhibits GSNOR, whereas *S*-sulfenylation, at Cys-284, negatively regulates its activity, demonstrating direct crosstalk between RNS and ROS signaling pathways.^84^ Identifying and engineering alleles with altered PTM sensitivity allows plants to adjust to changing redox conditions under hypoxia without disrupting essential GSNOR functions. Furthermore, GSNOR does not operate alone. Several other enzymes participate in NO/GSNO regulation and may serve as additional targets of manipulation. AKRs are strongly upregulated in GSNOR-deficient plants and can degrade GSNO in the absence of GSNOR.^91^ The cytoplasmic TRX*h*5 system, acting as a denitrosylase, can cleave GSNO and denitrosylate protein SNOs.^65^^,^^125^ Additionally, ROG1 acts as a transnitrosylase that specifically *S*-nitrosylates GSNOR1 at Cys-10, impacting its stability and promoting its degradation.^104^ A multigene stacking strategy, which combines beneficial alleles in GSNOR and its regulators, may improve NO scavenging. Interestingly, while GSNOR is a single-copy gene in Arabidopsis, it is duplicated in certain higher plants such as legumes (e.g., two copies in *Lotus japonicus*), potentially contributing to divergent stress responses.^93^ For instance, these paralogs exhibit tissue-specific expression patterns, such as *LjGSNOR2*, which is highly expressed in nodules, suggesting specialized functions that enhance adaptation.^93^ The engineering of GSNOR can lead to promising results in terms of stress tolerance, particularly under hypoxia. However, an approach to engineer only GSNOR might not be sufficient since GSNOR activity and stability are regulated in multiple manners, necessitating a multidirected approach to fine-tune activity.^27^^,^^97^

Table 1 summarizes the known GSNOR regulation, its potential function and provides a strategy for regulating GSNOR.

**Table 1.** Potential approaches to engineer or modify GSNOR expression and activity.

Target	Function	Strategy
*GSNOR* expression	NO buffering	Stress or tissue specific promoters *cis*-regulatory elements
GSNOR’s PTM sites	Resistance to PTMs Prevention of degradation via autophagy	Mutagenesis of key residues (e.g. Cys-10 and Cys-284)
Interaction of TRX and GSNOR	Regulation of GSNOR via denitrosylation	Enhanced interaction between GSNOR and TRX for improved activity restoration
ROG1	Promoting GSNOR degradation	Knockdown or knockout during specific stress phases to stabilize GSNOR
AKRs	Compensatory GSNO degradation when GSNOR is impaired	Overexpression in crops with limited GSNOR activity
Natural variation of *GSNOR* alleles across different species with contrasting flooding tolerance	Functional diversity affecting NO regulation and hypoxia response	Genome-Wide Association Studies (GWAS), allele mining, introgression into target crops

## Concluding remarks and open questions

**Figure 2.**
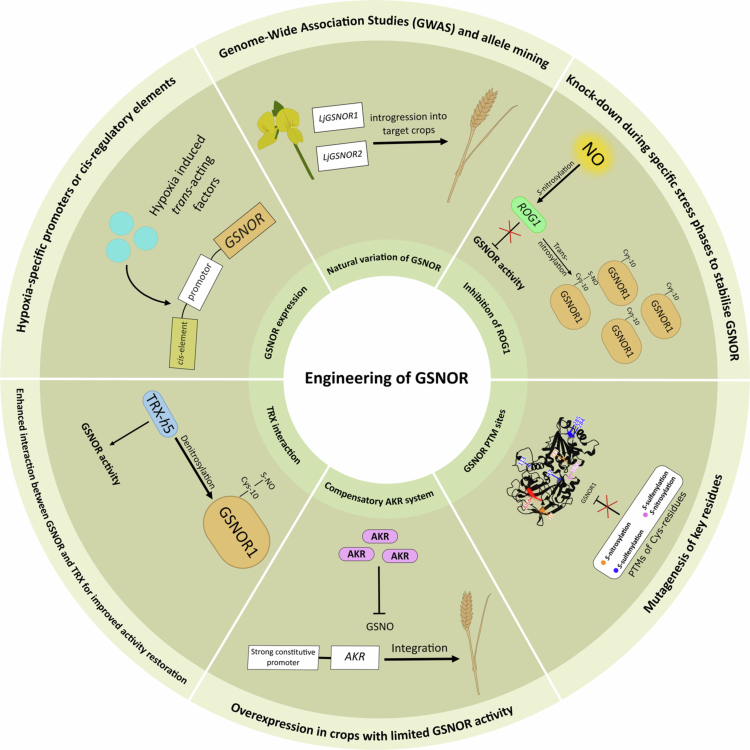
Possible strategies for manipulating or modulating *GSNOR* expression and activity to improve the hypoxia tolerance of crops. Genetic strategies include genome-wide association studies (GWAS) and allele mining to identify beneficial variants, as well as targeted knockdown of the *Repressor of GSNOR1* (*ROG1*) to enable GSNOR activity during specific flooding phases. Cys residues of GSNOR can be modified to prevent PTMs such as *S*-nitrosylation of Cys-10 and the associated Autophagy-related protein 8 (ATG8)-mediated degradation. Compensatory pathways, including those involving the overexpression of aldo-keto reductases (AKRs), may help maintain cellular NO homeostasis when GSNOR activity is reduced. Modulation of protein‒protein interactions, for example, with thioredoxin (TRX), can be optimized to increase GSNOR activity during specific phases of hypoxia. Transcriptional regulation can be achieved via stress- or tissue-specific promoters, *cis*-regulatory elements, and hypoxia-inducible *trans*-acting factors that bind to these elements. The protein structure of the GSNOR1 monomer from Arabidopsis was obtained from Alphafold 3.^112^

GSNOR has evolved from a NO scavenger to a key player in the plant hypoxia network, coordinating redox homeostasis, posttranslational control and cell survival. Recent evidence suggests that it is a dynamic regulatory node integrating NO turnover, autophagy and redox signaling. The presence of developmental defects in both the overexpression and loss-of-function mutants highlights the importance of precise temporal and spatial regulation of GSNOR function rather than overall abundance. Understanding GSNOR regulation during hypoxia extends beyond enzyme kinetics and defines how plants control NO-mediated signaling in accordance with oxygen levels. PTMs, gene copy variation, and protein–protein interactions reveal a broader regulatory network in which GSNOR acts in concert with other partners, such as TRX*h*5, QSOX1, AKRs, and ROG1. Therefore, NO homeostasis is multifaceted and not the outcome of a single enzyme. A future challenge will be the translation of this molecular understanding into engineering frameworks. Precise modulation of GSNOR using stress-inducible promoters, PTM-resistant alleles or synthetic regulons could potentially increase tolerance without the developmental costs associated with established mutants. Combining GSNOR-targeted strategies with existing hypoxia-responsive pathways, such as the ERFVII and PCO networks, would create crops with flexible and adaptive stress responses. As climate change progresses, understanding the precise mechanism of regulation and function of GSNOR under hypoxia will be a promising approach for improving plant survival under flooding and can strengthen the molecular roadmap to a more secure global food supply (Figure 2).

Long-standing questions in plant NO biology, as well as hypoxia signaling and tolerance that could be addressed by manipulating or investigating the regulation of GSNOR activity include:

1. How is GSNOR activity regulated during flooding? One major unresolved question is how plants balance the immediate need to buffer excess NO with the longer-term requirement to reduce GSNOR activity and activate anaerobic survival pathways. Understanding how these opposing priorities are coordinated in time and across tissues remains a key research challenge.
2. How redundant is NO detoxification in planta? Although enzymes such as AKRs can partially compensate for the loss of GSNOR activity, how plants fine-tune NO and GSNO levels under fluctuating stress intensity remains unclear. This is especially relevant given the reversible regulation of GSNOR by proteins such as TRX*h*5 and QSOX1, which suggests a highly dynamic, layered control mechanism.
3. Does gene copy number shape stress response capacity? GSNOR is encoded by a single gene in Arabidopsis but by multiple copies in legumes. Whether this genomic architecture influences hypoxia tolerance is not yet known. A key question is whether increasing the GSNOR gene dosage in single-copy species could increase their resilience to flooding without compromising development or immune signaling.
4. Can synthetic biology overcome native regulatory trade-offs? A promising frontier is the use of orthogonal gene circuits or nonnative NO scavenging systems. Whether such tools can be deployed in a spatially and temporally controlled manner, without unintended effects on growth, signaling, or immunity, is still unknown.
5. What role does GSNO play in regulating ERFVII transcription factors? While the roles of NO and oxygen in controlling ERFVII stability are becoming clearer, the specific molecular mechanism by which GSNO contributes to this regulation remains unresolved.
6. Are NO and GSNO functionally redundant or mechanistically distinct? It is increasingly plausible that GSNO has regulatory functions beyond serving as an NO reservoir. Whether GSNO can directly modulate ERFVII activity, or other hypoxia signaling components, independent of NO release remains an open and compelling research question.
